# Prognostic Value of Drug Targets Predicted Using Deep Bioinformatic Analysis of m6A-Associated lncRNA-Based Pancreatic Cancer Model Characteristics and Its Tumour Microenvironment

**DOI:** 10.3389/fgene.2022.853471

**Published:** 2022-04-25

**Authors:** Peng-Wei Cao, Lei Liu, Zi-Han Li, Feng Cao, Fu-Bao Liu

**Affiliations:** ^1^ Hepatopancreatobiliary Surgery, Department of General Surgery, The First Afliated Hospital of Anhui Medical University, Hefei, China; ^2^ NHC Key Laboratory of Study on Abnormal Gametes and Reproductive Tract (Anhui Medical University), Hefei, China; ^3^ Department of General Surgery, The Second Hospital of Anhui Medical University, Hefei, China

**Keywords:** pancreatic cancer, N6-methyladenosine, long non-coding RNA, tumour, tumor microenvironment

## Abstract

The role of N6-methyladenosine (m6A)-associated long-stranded non-coding RNA (lncRNA) in pancreatic cancer is unclear. Therefore, we analysed the characteristics and tumour microenvironment in pancreatic cancer and determined the value of m6A-related lncRNAs for prognosis and drug target prediction. An m6A-lncRNA co-expression network was constructed using The Cancer Genome Atlas database to screen m6A-related lncRNAs. Prognosis-related lncRNAs were screened using univariate Cox regression; patients were divided into high- and low-risk groups and randomised into training and test groups. In the training group, least absolute shrinkage and selection operator (LASSO) was used for regression analysis and to construct a prognostic model, which was validated in the test group. Tumor mutational burden (TMB), immune evasion, and immune function of risk genes were analysed using R; drug sensitivity and potential drugs were examined using the Genomics of Drug Sensitivity in Cancer database. We screened 129 m6A-related lncRNAs; 17 prognosis-related m6A-related lncRNAs were obtained using multivariate analysis and three m6A-related lncRNAs (*AC092171.5, MEG9, and AC002091.1*) were screened using LASSO regression. Survival rates were significantly higher (*p* < 0.05) in the low-risk than in the high-risk group. Risk score was an independent predictor affecting survival (*p* < 0.001), with the highest risk score being obtained by calculating the c-index. The TMB significantly differed between the high- and low-risk groups (*p* < 0.05). In the high- and low-risk groups, mutations were detected in 61 of 70 samples and 49 of 71 samples, respectively, with *KRAS*, *TP53*, and *SMAD4* showing the highest mutation frequencies in both groups. A lower survival rate was observed in patients with a high versus low TMB. Immune function HLA, Cytolytic activity, and Inflammation-promoting, T cell co-inhibition, Check-point, and T cell co-stimulation significantly differed in different subgroups (*p* < 0.05). Immune evasion scores were significantly higher in the high-risk group than in the low-risk group. Eight sensitive drugs were screened: ABT.888, ATRA, AP.24534, AG.014699, ABT.263, axitinib, A.443654, and A.770041. We screened m6A-related lncRNAs using bioinformatics, constructed a prognosis-related model, explored TMB and immune function differences in pancreatic cancer, and identified potential therapeutic agents, providing a foundation for further studies of pancreatic cancer diagnosis and treatment.

## Introduction

Pancreatic cancer is currently one of the most malignant tumours worldwide and is difficult to diagnose in early stages, leading to poor treatment outcomes and high mortality rates, thus posing a serious global public health risk. According to the 2020 global statistics, there are approximately 500,000 new cases of pancreatic cancer and 460,000 deaths each year (Sung et al., 2021). The main treatment modality is surgery supplemented with chemotherapy and immune-targeted therapy; however, the resectability rate using surgery is only 15–20%, and adjuvant treatments show limited effects. Although, Studies have shown that nab-paclitaxel and mFOLFIRINOX (Oxaliplatin + Irinotecan+5-Fluorouracil + Calcium folinate) can effectively improve the survival rate of pancreatic cancer patients (De Luca et al., 2018; Galvano et al., 2020). However, the overall 5-years survival rate of less than 8% (Shaib et al., 2016; Ying et al., 2016; Mcguigan et al., 2018). In recent years, numerous studies have shown that this may be related to tumor microenvironment (TME), copy number variation and tumor cell heterogeneity (Su et al., 2020). Studies have shown that CNV may be related to pancreatic cancer, which is beneficial to the early diagnosis of the disease (Fanale et al., 2013). N6methyladenosine (m6A) methylation is a widely occurring RNA modification, and is mainly regulated by methyltransferases, demethylases, and methylation reading proteins. It plays an integral role in tumor cells and cells of the TME such as immune cells, inflammatory cells, and endothelial cells (Sun et al., 2019; Liu et al., 2019). Studies have shown that m6A suppression can promote the proliferation and invasion of gastric cancer cells by activating the Wnt and PI3K-Akt signaling pathways Furthermore, Xia *et al* found that the m6A-related gene *METTL3* was highly expressed in pancreatic tumor cells, and METTL3 knockdown could inhibit the proliferation and migration of tumor cells (Xia et al., 2019). He Y *et al* found that the m6A eraser protein ALKBH5 can inhibit pancreatic cancer progression by reducing the methylation of long non-coding RNA (lncRNA) KCNK15-AS1, suggesting it may be a potential therapeutic target for pancreatic cancer (He et al., 2018). lncRNA can be classified as signalling, decoy, guide, and scaffold lncRNAs according to their functions. Aberrant expression of lncRNAs disrupts homeostasis in organisms and may drive or inhibit various cancers (Bhan et al., 2017). It also plays an important role in tumorigenesis and development (Schmitt and Chang, 2016). Downregulation of LINC01232 can inhibit the proliferation and spread of pancreatic cancer cells to improve prognosis (Li et al., 2019). N6-methyladenosine (m6A) is an epigenetic regulatory alteration of RNA (Sunwoo et al., 2015) that affects the processing, transport, and stability of lncRNAs through internal modifications and thus influences biological processes (Zhou et al., 2016). Yang X *et al* found that inhibiting the expression of lncRNA XIST by down-regulating the m6A-related protein METTL14 inhibited the proliferation and metastasis of colorectal cancer (Yang et al., 2020). In addition, it was shown that lung cancer associated transcript 3 (LCAT3), a new m6A-regulated lncRNA, can bind to Far Upstream Element Binding Protein 1 (FUBP1) activating MYC transcription and promoting lung cancer cell proliferation, invasion and metastasis (Qian et al., 2021). However, studies of m6A-related lncRNAs in pancreatic cancer have rarely been reported, especially studies of their molecular mechanism and role in prognosis. Therefore, this study aimed to screen m6A-related lncRNAs in pancreatic cancer cells and comprehensively analyze their potential role in clinical features, prognosis and TME. Wel also sought to construct a prognostic prediction model to explore possible molecular markers and drug targets point to provide a new research strategy for pancreatic cancer immunotherapy.

## Methods

### Collection and Processing of Pancreatic Cancer Data

We obtained clinical data from 182 patients with pancreatic cancer from The Cancer Genome Atlas (TCGA) database (https://portal.gdc.cancer.gov/), transcriptome profling FPKM data was sourced, and identifying information was redacted from all patients. A total of 178 patient samples was defined as a combined set, which was randomly divided into a test group, and training group. The data were normalised, processed, and analysed using R software 4.1.1 (The R Group for Statistical Computing, Vienna, Austria).

### Screening of m6A-Associated lncRNAs

We extracted lncRNA from FPKM data via human genome annotation date GTF files from Ensembl (http://asia.Ensembl.org) and identified 24 m6A-related genes from literatures, including writers (*METTL3*, *METTL14*, *METTL16*, *WTAP*, *VIRMA*, *ZC3H13*, *RBM15*, *RBM15B*), readers (*YTHDC1*, *YTHDC2*, *YTHDF1*, *YTHDF2*, *YTHDF3*, *HNRNPC*, FMR1, *LRPPRC*, *HNRNPA2B1*, *IGFBP1*, *IGFBP2*, *IGFBP3*, and *RBMX*), and erasers (*FTO* and *ALKBH5*). The m6A genes and lncRNAs in the samples were evaluated using Pearson correlation analysis, with pre-set criteria of correlation> 0.4 and *p* < 0.001.

### Modelling and Evaluation of m6A-Associated lncRNA Gene Markers

Univariate COX regression analysis was performed to screen LncRNAs associated with tumor prognosis using canonical m6A-related LncRNAs and clinical data. Then, the prognosis-related LncRNAs were further subjected to least absolute shrinkage and selection operator (LASSO) and multivariate COX regression analysis to calculate the risk score (RS) of each sample, and construct a prognostic model. Uni/multivariate COX analysis was performed based on patient clinical data and RS, and “survcomp” software package used for calculating the c-index index for evaluating the best prediction of the model. Next, the samples were divided into two groups according to the characteristics and coefficients: high- and low-risk groups, and receiver operating characteristic (ROC) curves were generated using the “survival” and “survivalROC” packages in R software. These ROC curves were used to analyse the 1-, 3-, and 5-years survival rates of patients and assess the accuracy of genetic characteristics for predicting survival. The “pheatmap” package was used to draw a heatmap of lncRNA of affecting prognosis in train and test groups. The “survcomp” package was used to calculate the c-index for assessing the best model prediction.

### Assessment of Tumour Immune Microenvironment and Mutational Load and Potential Drug Screening Using m6A-Associated lncRNA Models

To evaluate the relative immune function differences between high/low risk groups, we downloaded the immune set files and used the “limma, GSVA, GSEABase, pheatmap, reshape2” package to determine and visualise the correlations of high- and low-risk groups with individual immune checkpoints. Tumor Immune Dysfunction and Exclusion (TIDE) was downloaded at http://tide.dfci.harvard.edu/login/ to print documents. The TIDE scores of high- and low-risk samples were calculated and visualised using “limma, ggpubr” in R. Differences in tumor mutational burden (TMB) and somatic mutations in samples were compared between high-risk and low-risk groups using “maftools” in R. According to the level of TMB, patients were divided into high/low-TMB group, and Kaplan-Meier survival analysis was performed. The Genomics of Drug Sensitivity in Cancer database (https://cancerrxgene.org) is used for large-scale drug screening. Combined with genomic analysis, differential responses to chemotherapeutic agents in tumor patients in high/low risk groups was systematically determined. Using the Genomics of Drug Sensitivity in Cancer database, the half-maximal inhibitory concentration (IC_50_) of drugs commonly used for pancreatic cancer tumours was calculated to assess the clinical application of this model in the treatment of pancreatic cancer. We then used Wilcoxon signed-rank test to compare the difference in IC_50_ between the high- and low-risk groups. To visualise the data, box plots were drawn using “PRrophytic” and “ggplot2” in R.

## Results

### m6A-Associated lncRNAs and Their Prognostic Values

**FIGURE 1:**
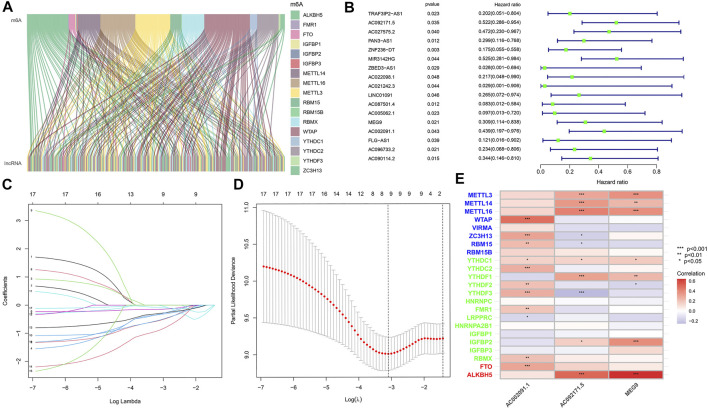
**(A)** Co-expression of m6A regulatory genes and related lncRNA. **(B)** Cox univariate analysis of m6A regulatory gene. **(C)** The tuning parameters of OS-related proteins to cross-verify the error curve. **(D)** Perpendicular imaginary lines to calculating the minimum criteria. **(E)** Correlation heatmap of 3 m6a associated lnCRNAs.

The expression profiles of 24 common m6A genes and 14,056 lncRNAs were obtained by analysing 178 pancreatic adenocarcinoma samples from TCGA. Using Pearson correlation analysis, we screened 129 m6A-associated lncRNAs (Figure 1A), which were used to derive 17 m6A-associated lncRNAs with prognostic value via co-expression analysis and univariate Cox regression analysis, the results of which are shown in the following figure (Figure 1B).

### TCGA Cohort-Based m6A-Associated lncRNA Model Construction

**FIGURE 2:**
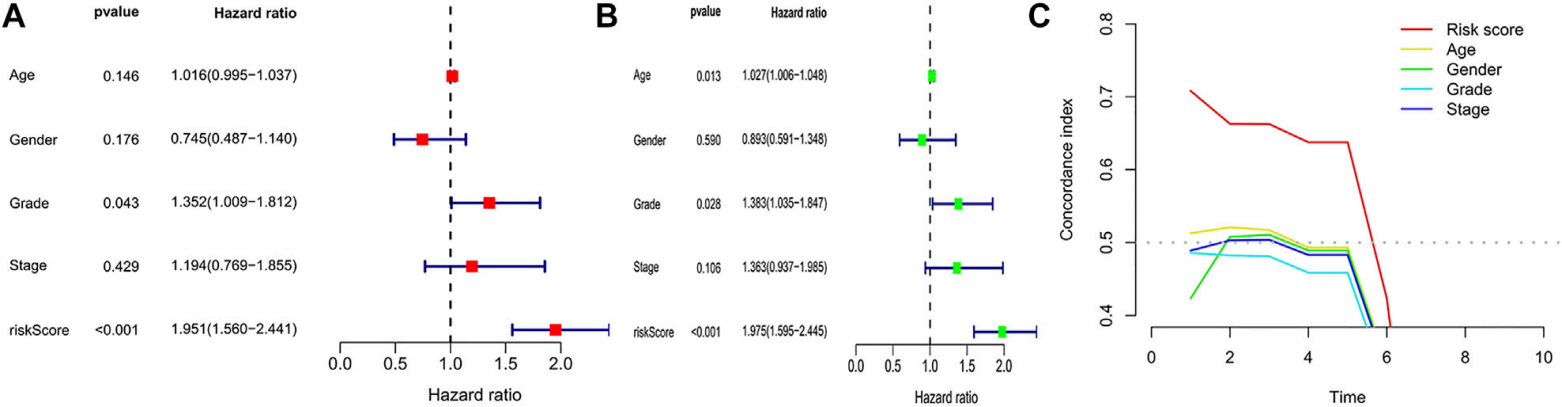
**(A)** Cox univariate analysis. **(B)** Cox multivariate analysis. **(C)** c-index analysis curve.

**FIGURE 3:**
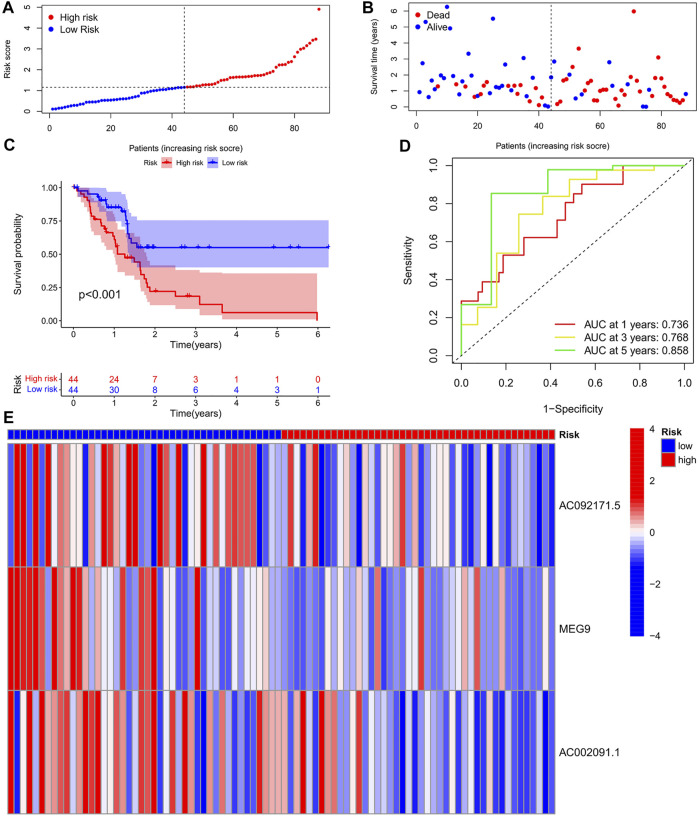
**(A)** Survival status of patients in different groups in Training cohort. **(B)** Rank of prognostic index and distribution of groups based on m6A-lncRNA prognostic signature risk scores in training cohort. **(C)** Survival status of patients in different groups in Training cohort. **(D)** Receiver operating characteristic (ROC) curves of the m6A-lncRNA prognostic signature for predicting the 1-year, 3-years, and 5-years survival in Train cohort. **(E)** heatmap of the m6A-lncRNA in Train cohort.

To eliminate covariate collinearity and avoid overfitting of the prognostic model, LASSO regression analysis was performed for the 17 differentially expressed m6A-associated lncRNAs with prognostic value to calculate the hazard coefficient for each LncRNA. 9 prognostic related lncRNAs were retained based on minimum partial likelihood deviance LncRNA (Figures 1C,D). Ultimately, m6A-lncRNAs were used to determine a prognostic model of patients with pancreatic adenocarcinoma based on the expression values of three lncRNAs (*AC092171.5, MEG9,* and *AC002091.1*) (Figure 1E). The genes showing high expression levels were *AC092171.5* [hazard ratio (HR) = 0.439, 95% confidence interval (CI) = 0.197–0.976)], *MEG9* (HR = 0.309, 95% CI = 0.114–0.838), *AC002091.1* (HR = 0.522, 95% CI = 0.288–0.954). The Cox coefficients of the three lncRNAs were used for modelling to calculate the prognostic risk scores for each patient in TCGA cohort as follows: (1.20237572230772 × expression level of *AC092171.5*) - (0.975350163451454 × expression level of *MEG9*)—(1.49408312354077 × expression level of *AC002091.1*). Multivariate Cox regression analysis, Cox univariate analysis and c-index curve showed that the risk scores were significantly associated with survival and independent of clinical parameters (Figures 2A,B,C). Subsequently, the patient in the test group were divided into high- and low-risk groups based on the median risk scores (Figures 3A,B). In the high-risk group, the expression levels of *AC092171.5*, *MEG9*, and *AC002091.1* were lower Compared to low-risk group (Figure 3E). According to the survival curves, the overall survival rate was lower in the high-risk subgroup than in the low-risk subgroup (Figure 3C). The ROC curves showed AUC values of 0.710, 0.745, and 0.838 at 1, 3, and 5 years, respectively (Figure 3D). Therefore, the time-dependent ROC curves validated the prognostic value of the risk score.

### Validation of Test Group-Based m6A-lncRNA Model

**FIGURE 4:**
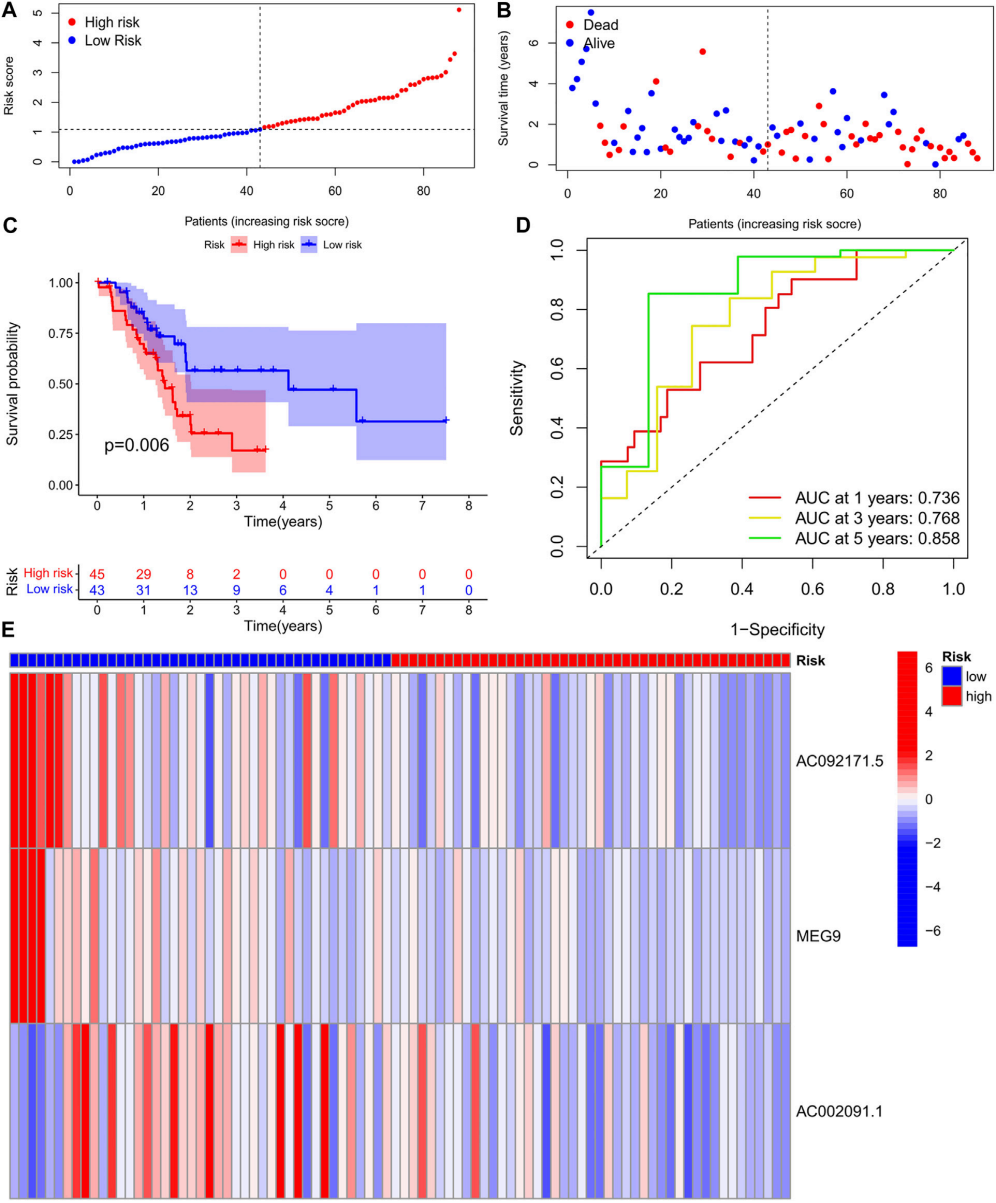
**(A)** Survival status of patients in different groups in test cohort. **(B)** Rank of prognostic index and distribution of groups based on m6A-lncRNA prognostic signature risk scores in training cohort. **(C)** Survival status of patients in different groups in Test cohort. **(D)** Receiver operating characteristic (ROC) curves of the m6A-lncRNA prognostic signature for predicting the 1-year, 3-years, 5-years survival in Test cohort. **(E)** Heatmap of the m6A-lncRNA in Test cohort.

To validate the prediction reliability of the training group m6A-lncRNA, the 88 samples in the test group were divided into high- and low-risk subgroups based on their median risk scores, which were calculated as described for the training group cohort (Figures 4A,B). In the risk curve, the survival rate of the high-risk gene group was lower than that of the low-risk gene group (Figure 4C). In the heat map, the expression of risk genes was similar to that in the training group (Figure 4E). In the survival curve, the survival time of the high-risk gene group was significantly shorter than that of the low-risk gene group. The AUC values at 1, 3, and 5 years in the time-dependent ROC curve were 0.688, 0.746, and 0.919, respectively (Figure 4D).

### Tumour Mutation Load Analysis

**FIGURE 5:**
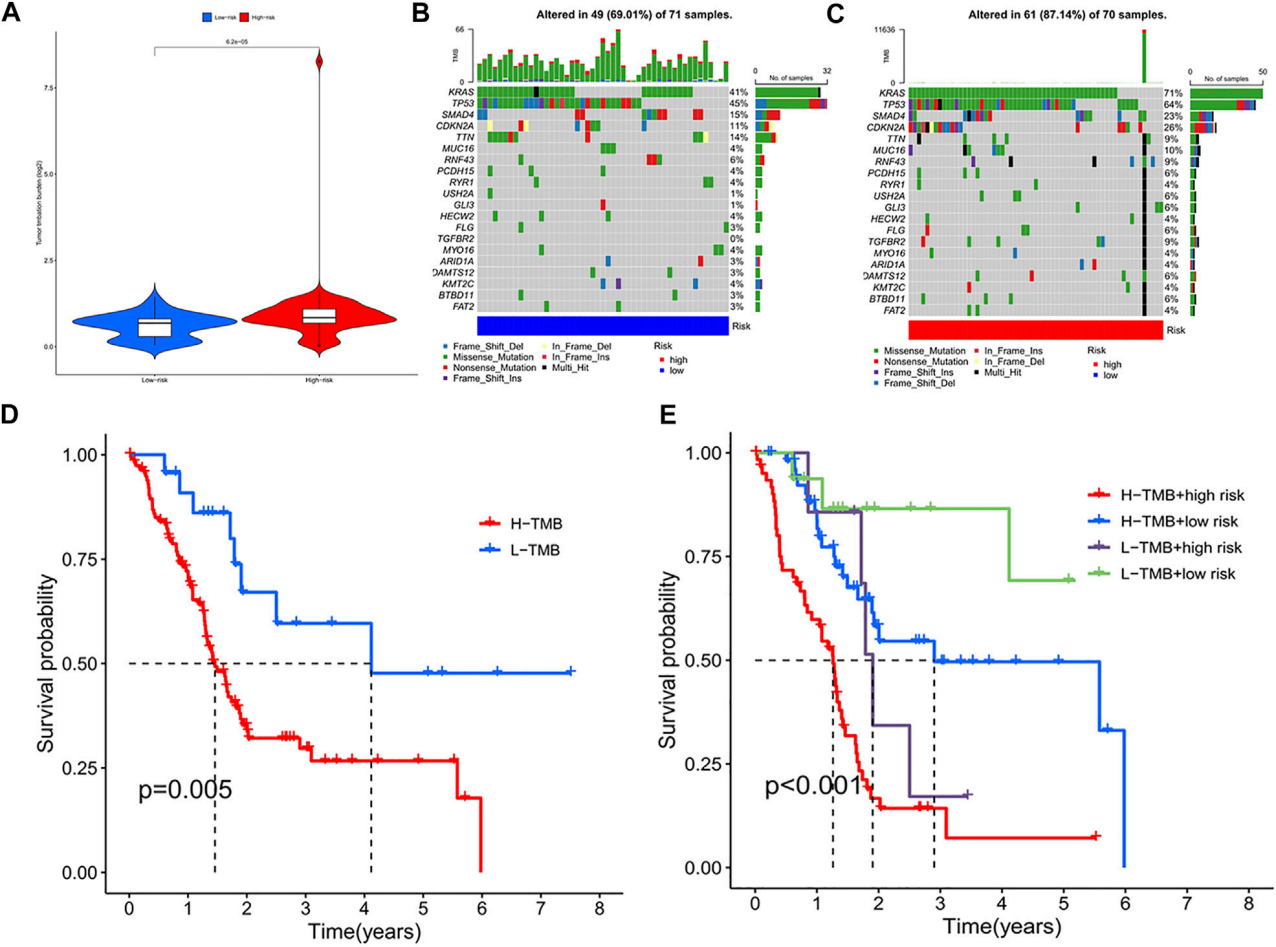
**(A)** TMB violin plot of high and low risk group. **(B)** High-risk group mutant gene waterfall plot. **(C)** Low-risk group mutant gene waterfall plot. **(D)** Kaplan-Meier curve of H-TMB and L-TMB. **(E)** Kaplan-Meier curve of TMB + Risk.

The tumor mutational burden (TMB) indices of high- and low-risk genes were calculated separately; as shown in the violin plot (Figure 5A), the TMB of differed for high- and low-risk genes (*p* < 0.05), with a higher TMB in the high-risk group. The waterfall plot shows the top 30 mutation frequencies. In the high-risk group (Figure 5B), mutations were detected in 61 of 70 samples; *KRAS* (71%), *TP53* (64%), and *SMAD4* (23%) showed the highest mutation frequencies. In the low-risk group (Figure 5C), mutations were detected in 49 of 71 samples, with *KRAS* (41%), *TP53* (45%), and *SMAD4* (15%) showing the highest mutation frequencies. As shown in the figure (Figure 5D), the survival rate of patients with a high TMB was lower than that of patients with a low TMB. Additionally, the mutation frequency of high-risk genes was greater than that of genes in the low-risk group, and the survival rate of the high-TMB + high-risk gene group was lowest, followed by the low-TMB + high-risk gene, high-TMB + low-risk gene, and low-TMB + low-risk gene groups (Figure 5E).

### Differences in Immune Function

**FIGURE 6:**
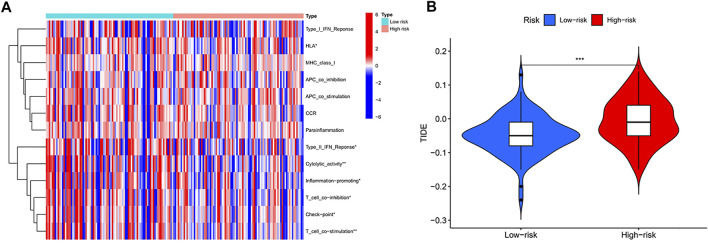
**(A)** Comparison of 13 immune-related functions between high-risk group and low-risk group in the TCGA cohort (*p*-values were showed as: NS, not significant; ∗*p* < 0.05, ∗∗*p* < 0.005,∗∗∗*p* < 0.001). **(B)** Violin plot of immune escape (*p*-values were showed as: ∗∗∗*p* < 0.001)

We observed significant differences in immune-related functions between the high- and low-risk groups (Figure 6A). As a practical tool for assessing anti-tumour immunity, HLA was significantly lower in the high-risk subgroup, indirectly indicating weaker HLA function in this subgroup; TypeII IFN Reponse was lower in the high-risk group than in the low-risk group. In addition, Cytolytic activity, Inflammation-promoting, T cell co-inhibition, Check-point, and T cell co-stimulation showed lower expression in the high-risk group than in the low-risk group. As shown in the figure (Figure 6B), the two groups were significantly different (*p* < 0.001), with higher TIDE scores in the high-risk than in the low-risk, indicating that the low-risk group has lower immune evasion potential and that immunotherapy has lower efficacy in the high-risk group.

### Screening for Potential Drugs

**FIGURE 7:**
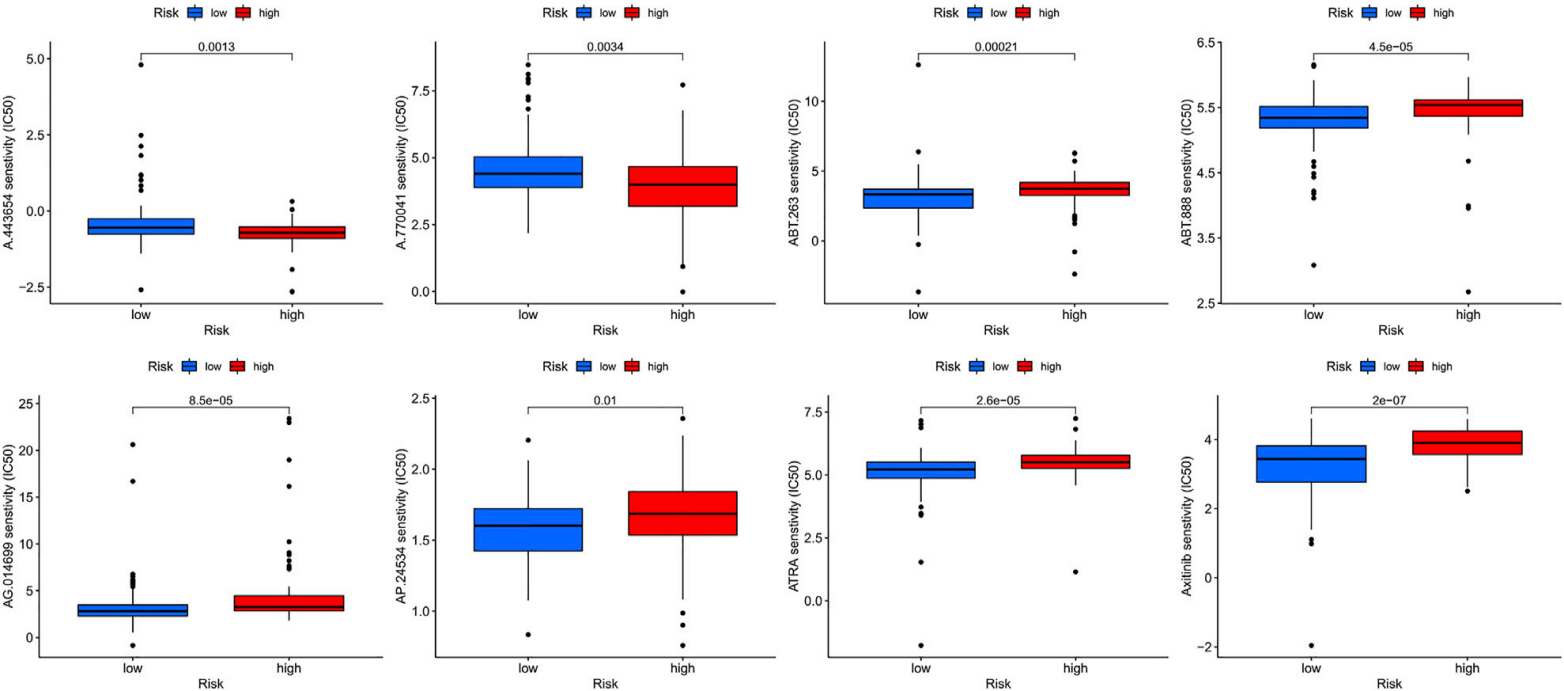
The sensitivity (IC50) of ABT.888, ATRA, AP.24534, AG.014699, ABT.263, axitinib, A.443654, and A.770041 between high and low risk groups.

Screening revealed eight potential therapeutic drugs which showed different sensitivities between high- and low-risk groups (*p* < 0.05): For ABT.888, ATRA, AP.24534, AG.014699, ABT.263, and axitinib, the IC_50_ in the low-risk group was less than that in the high-risk group (Figures 7A–E), indicating that patients in the low-risk group were more sensitive to the drugs. For A.443654 and A.770041, the IC_50_ in the low-risk group was lower than that in the high-risk group, indicating that patients in the high-risk group are more sensitive to these drugs (Figures 7F–J).

## Discussion

Pancreatic cancer is a common and highly malignant tumour of the digestive system that is currently difficult to detect, diagnose, and treat early because of its unique biology. The main therapeutic options for pancreatic cancer include surgery, chemotherapy, and radiotherapy (Murphy et al., 2018; De Dosso et al., 2021). However, only approximately 20% of patients can be treated with surgery (Murphy et al., 2018; De Dosso et al., 2021). Studies (Siegel et al., 2021) have shown that the median survival time after surgery is only 24–30 months. Therefore, it is important to find a reliable biomarker for determining the prognosis of pancreatic cancer. LncRNAs play important roles in biological processes by regulating gene expression and influencing various aspects of these processes such as transcriptional regulation, gene imprinting, and chromatin remodelling (Ponting et al., 2009; Kanduri, 2011). In previous studies, m6A-related lncRNAs were shown to determine the prognosis of lung adenocarcinoma and low-grade glioma (Lin et al., 2016; Wang et al., 2021). Therefore, m6A-related lncRNAs show potential as valuable targets for the diagnosis and treatment of pancreatic cancer. In this study, we integrated m6A-related lncRNAs from the Ensembl database and m6A-related lncRNAs from TCGA database and their corresponding clinical data, which were used to construct a prognostic model of m6A-associated lncRNAs and internal validation. The relationship between various genes and the immune microenvironment was explored and screened to identify relevant drugs.

We performed a series of bioinformatics analyses to develop an m6A-associated lncRNA model for determining the prognosis of pancreatic cancer. Three lncRNAs (*AC092171.5, MEG9, and AC002091.1*) were screened for subsequent analysis. Guyugang et al. (Guo et al., 2021) found that *AC092171.5* was expressed at lower levels in patients with high-risk lung adenocarcinoma, and Wei et al. (Wei et al., 2021) found that patients with high *AC002091.1* expression in osteosarcoma had a better prognosis. In our study, *MEG9*, *AC092171.5*, and *AC002091.1* were highly expressed in low-risk patients; these three lncRNAs may suppress pancreatic adenocarcinogenesis. In the training group, the survival of patients in the high-risk group was shorter than that in the low-risk group, whereas the test group showed consistent results. Taken together, these results confirm that our m6A-associated lncRNA model can accurately predict survival time in this population.

In addition, m6A methylation modifications play integral roles in innate and adaptive immune responses, which may be useful for further studies of pancreatic cancer pathogenesis. Immune cells in the TME of pancreatic cancer are in a state of quantity and functional imbalance, and have clearly defective anti-tumour functions, resulting in a unique TME (Zheng et al., 2017). The TME of pancreatic cancer is characterised by dense fibrosis and massive inflammatory cell infiltration, which prevent infiltration of effector cells such as T cells and natural killer cells into the tumour. Abundant stromal and inflammatory cells in the TME of pancreatic cancer can promote immune cell recruitment and activation. Large numbers of fibroblasts easily lead to scar-like tissue deposition, creating a physical barrier that blocks further cytotoxic T cell infiltration into the tumor, thereby contributing to immune escape in pancreatic cancer (Monteran and Erez, 2019; Johnson and June 2017). McGranahan et al. (McGranahan et al., 2017) showed that reduced or absent HLA-1 expression prevents a presented antigen from being recognised by T cells, thus causing immune evasion of the tumour. In our study, HLA function was lower in the high-risk group than in the low-risk group, suggesting a greater capacity for immune evasion in the high-risk group. In addition, tumour surface antigens can bind to effector cell immune checkpoint molecules, which can promote *in vivo* effector T cell hypofunction or apoptosis to promote immune evasion by tumours (Kalbasi and Ribas, 2020). We observed lower immune checkpoint function in the high-risk group than in the low-risk group, which may also contribute to poor prognosis.

In recent years, immunotherapy has shown initial success in treating malignant tumours and good efficacy towards melanoma, lung cancer, and other malignant tumours (Ohaegbulam et al., 2015). Exploring potentially mutated genes in pancreatic cancer can facilitate diagnosis and rational treatment selection. In our study, there was a clear difference in TMB between high and low risk groups. Interestingly, there was a large bias in the high risk group, which may be related to tumor TME changes, external carcinogens, different detection methods, and/or tumor heterogeneity (Merino et al., 2020; Hatakeyama et al., 2020). Besides, KARS and TP53 showed high mutation rates in both the high- and low-risk groups. Cullis et al. (Cullis et al., 2018) showed that mutations in KRAS are an important cause of the immunosuppressive state of tumours, as mutations in KRAS impair recognition of pancreatic cancer cells by T cells to result in immune evasion (Cullis et al., 2018). In addition, immunosuppression in the TME is related to mutations in KRAS. A related study (Merz et al., 2021) showed that mutations in the TP53 gene are common in tumours and affect the recruitment and activity of T cells, thus also leading to immune evasion (Blagih et al., 2020). These m6A-associated lncRNA gene mutations are closely associated with immune activity in the TME, suggesting an interaction between m6A modifications and tumour immunogenomics.

Our study had some limitations. First, this study was limited to TCGA database, and no further validation was performed using external databases. Second, our results must be validated in further experiments, and we have collected relevant clinical samples to perform this validation. Finally, animal and clinical trials are needed to explore the therapeutic effects of the predicted drugs on pancreatic cancer. However, our study provides a foundation for further exploration of biomarkers and immune mechanisms for diagnosing and treating pancreatic cancer based on m6A-related lncRNAs.

## Conclusion

We screened 17 differentially expressed m6A-associated lncRNAs with prognostic value based on 182 pancreatic cancer samples and identified three lncRNAs based on expression to construct and validate an m6A-lncRNA model. We found that these genes are correlated with the immune function characteristics of the TME and tumour conformity and calculated immune evasion scores for high- and low-risk groups. Finally, we screened relevant drugs to further identify tumour immunophenotypes to guide clinical applications.

## Data Availability

Publicly available datasets were analyzed in this study. This data can be found here: https: //portal. gdc. cancer.gov/ http: //asia.Ensembl.org http: //tide.dfci.harvard.edu/login/ https: //cancerrxgene.org.

## Abbreviations

N6-methyladenosine; m6A, Non-coding RNA; lncRNA, Least absolute shrinkage and selection operator; LASSO, Tumor mutational burden; TMB, Tumour immune microenvironment; TME, The Cancer Genome Atlas; TCGA, Half-maximal inhibitory concentration; IC50.
